# CFTR_TL: Transfer Learning-Enhanced Prediction of CFTR ATP Binding Sites with Multi-Window Convolutional Neural Networks

**DOI:** 10.1021/acsomega.5c06644

**Published:** 2025-11-19

**Authors:** Yu-Cheng Lee, Yan-Yun Chang, Wei-En Jhang, Van The Le, Sin-Siang Wei, Yu-Yen Ou

**Affiliations:** † Department of Computer Science and Engineering, 34895 Yuan Ze University, Chung-Li 32003, Taiwan; ‡ Graduate Program in Biomedical Informatics, Yuan Ze University, Chung-Li 32003, Taiwan

## Abstract

The Cystic Fibrosis Transmembrane Conductance Regulator (CFTR) protein, crucial for chloride ion transport across epithelial cells, requires ATP binding for proper function. Mutations within CFTR’s nucleotide-binding domains (NBDs) disrupt this process, leading to cystic fibrosis. Accurately predicting ATP binding sites within CFTR is essential for understanding its function and developing targeted therapies. However, the unique nature of CFTR as an ion channel within the ATP-binding cassette (ABC) transporter family poses challenges for general prediction methods. We present CFTR_TL, a novel approach that leverages transfer learning to enhance ATP binding site prediction in CFTR. Our approach involves training a base model on a broad data set of ATP-binding proteins, followed by fine-tuning with a data set enriched in ABC transporters, capitalizing on their functional similarity to CFTR. By utilizing a multiwindow convolutional neural network (CNN) to effectively capture spatial patterns, CFTR_TL achieves superior performance compared to traditional prediction methods. The resulting model demonstrates improved accuracy and specificity in identifying critical binding residues within CFTR. This approach not only provides a powerful tool for CFTR research but also offers a generalizable framework for tailoring prediction models in other protein families.

## Graphical abstract

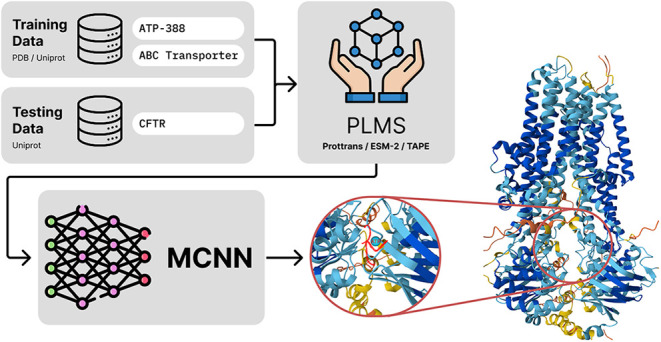

## Introduction

Cystic fibrosis (CF) is a severe genetic disorder arising from mutations in the CFTR gene, which encodes the Cystic Fibrosis Transmembrane Conductance Regulator (CFTR) protein.^1–3^ CFTR functions as an ion channel, facilitating chloride ion transport across epithelial cell membranes. The proper functioning of CFTR is essential for maintaining the balance of salt and water on epithelial surfaces, particularly in the lungs, pancreas, and digestive system. The activity of CFTR is critically dependent on the binding and hydrolysis of adenosine triphosphate (ATP) at its two nucleotide-binding domains (NBDs), which regulate channel opening and closing as illustrated in Figure 1. Mutations within these NBDs that disrupt ATP binding lead to the characteristic symptoms of CF, including thick mucus accumulation in the lungs and pancreas, resulting in respiratory infections, pancreatic insufficiency, and other complications.

**Figure 1:**
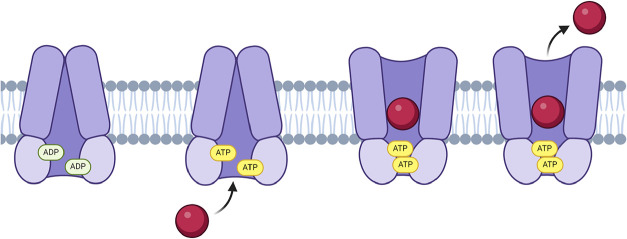
Diagram of the process of CFTR protein channel opening after ATP binding.

Given the central role of ATP binding in CFTR function and dysfunction, accurate identification of ATP binding sites within the protein is paramount for understanding the molecular basis of CF and for developing targeted therapies.^4,5^ While general ATP binding site prediction methods have proven useful for a wide range of proteins, they may not be optimally suited for CFTR due to its unique characteristics as an ion channel within the broader ATP-binding cassette (ABC) transporter family. The structural and functional nuances of CFTR, including its intricate gating mechanisms and dynamic conformational changes, necessitate a more refined and tailored approach for accurately identifying ATP binding sites as illustrated in Figure 2.

**Figure 2:**
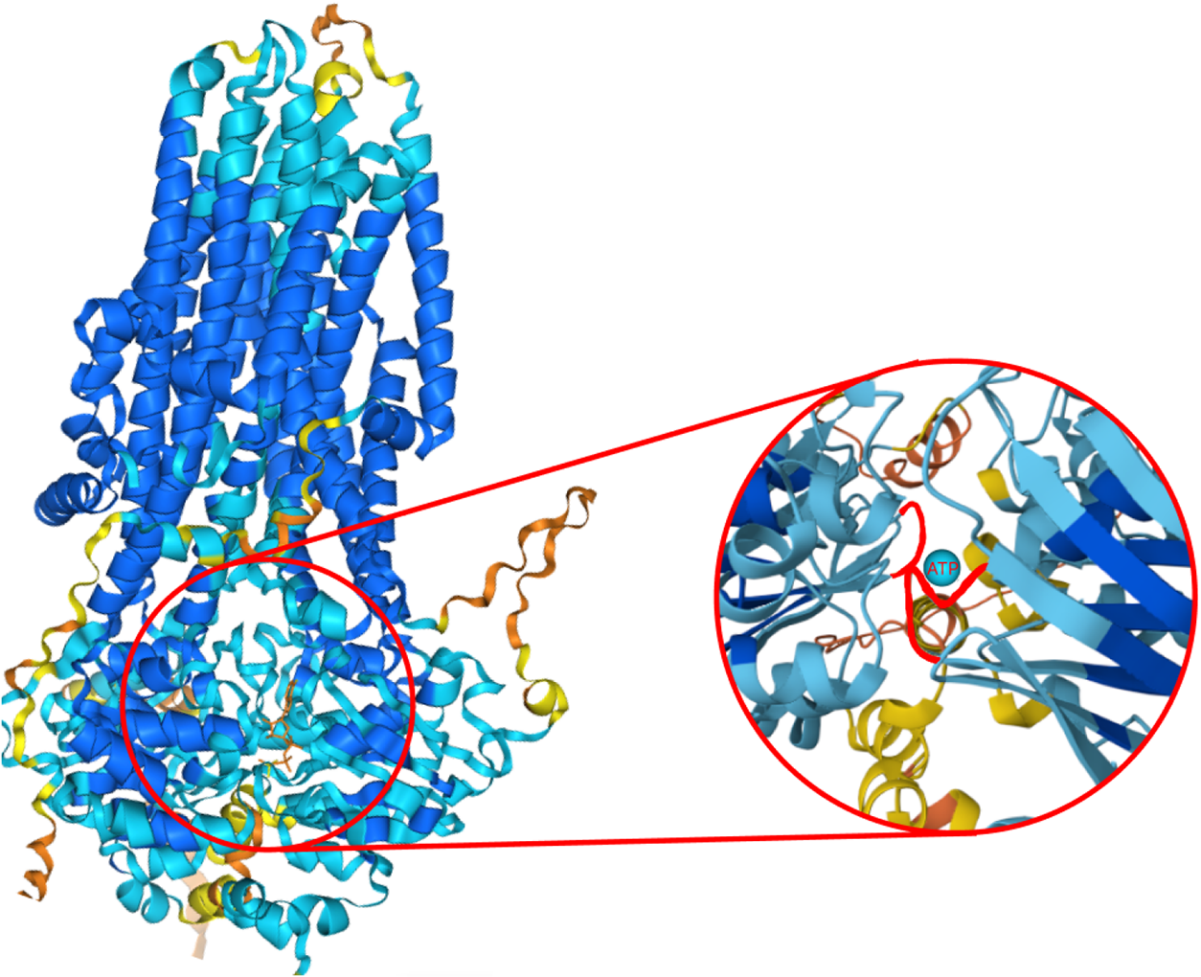
Red areas show where the CFTR Protein binds to ATP.

Recent advances in protein sequence analysis have ushered in a new era of computational biology, particularly with the advent of pretrained language models (PLMs) and multiscan convolutional neural networks (CNNs). PLMs, trained on vast protein data sets, excel at capturing intricate biological signals and evolutionary patterns encoded within amino acid sequences. Models like ESM-2 ^6^ and ProtTrans^7^ have demonstrated remarkable accuracy in predicting protein structures, functions, and interaction sites, eliminating the need for laborious feature engineering. This capability is especially valuable for studying complex proteins like CFTR, where traditional experimental methods can be time-consuming and expensive.^8,9^

Complementing PLMs, multiwindow CNNs have revolutionized structural bioinformatics.^10–13^ By analyzing protein structures across multiple scales and orientations, these networks capture a wealth of spatial and textural information, proving particularly effective for identifying complex features like ATP binding sites. Multiwindow CNNs, in particular, leverage parallel convolutional filters to scan protein surfaces from different orientations and resolutions, effectively capturing fine-grained structural details essential for characterizing complex functional sites. This synergy between cutting-edge deep learning approaches not only accelerates fundamental discoveries in CFTR research but also holds immense potential for therapeutic advancements, ultimately contributing to the development of precision medicine strategies for cystic fibrosis treatment.

In this study, we present CFTR_TL, a novel approach that synergistically integrates the strengths of PLMs and multiscan CNNs to achieve enhanced ATP binding site prediction specifically for CFTR. We leverage transfer learning to tailor our model to this unique protein. First, we train a base multiwindow CNN model on a diverse data set of ATP-binding proteins, allowing it to learn generalizable features associated with ATP binding. Subsequently, we fine-tune this model using a data set enriched with ABC transporter proteins, exploiting their functional similarity to CFTR and enabling the model to specialize its predictive capabilities for this critical protein family.

Although high-resolution crystal and cryo-EM structures have precisely mapped the ATP-binding sites of CFTR’s nucleotide-binding domains (NBD1 and NBD2), these structures provide only static snapshots and cannot fully address the needs of variant analysis or high-throughput screening. More than 2,000 CFTR mutations have been reported, and many disease-associated variants occur in or near the NBDs, potentially altering ATP interactions either directly or indirectly. Experimental determination of structures for every clinically relevant variant or ortholog is resource-intensive and often impractical, leaving large gaps in our understanding of how sequence changes affect ATP binding. To complement these structural studies, our CFTR_TL framework offers a rapid, sequence-based prediction tool that can evaluate ATP-binding sites in novel or understudied mutants and CFTR-related proteins. By enabling high-throughput assessment of binding potential from sequence data alone, CFTR_TL helps prioritize experimental efforts and supports personalized medicine approaches where structural information is unavailable or incomplete.

Through rigorous evaluation, we demonstrate that CFTR_TL significantly outperforms existing prediction methods, exhibiting improved accuracy and specificity in identifying key ATP binding residues within CFTR. Our findings highlight the immense potential of combining PLMs, multiscan CNNs, and transfer learning for enhancing bioinformatic predictions within specific protein families. This approach offers a generalizable framework that can be extended to other proteins with unique characteristics, paving the way for more accurate and targeted predictions across a wide range of biological contexts.

## Materials and Methods

### Data Collection

To develop and evaluate our CFTR-specific ATP binding site predictor, we carefully curated data sets encompassing both general ATP-binding proteins and those closely related to CFTR’s function. We started with the ATP-388 data set, a collection of 388 nonredundant ATP-binding protein chains from the Protein Data Bank.^14^ This data set, containing 5657 ATP-binding and 142,086 nonbinding residues, served as the foundation for our base model, enabling it to learn general patterns associated with ATP binding.

Recognizing the unique nature of CFTR as an ion channel within the ABC transporter family, we enriched our training data with a specialized data set. We retrieved 176 reviewed ABC transporter sequences from UniProt,^15^ specifically selecting entries annotated with ATP binding sites. This data set, comprising 1,842 ATP-binding and 142,578 nonbinding residues, allowed the model to learn features specific to the functional context of CFTR.

Finally, we included a dedicated CFTR data set consisting of 29 reviewed UniProt entries. These sequences contained 298 ATP-binding and 24,878 nonbinding residues, providing focused training data for refining our predictor. Both the ATP-388 and ABC transporter data sets were processed using CD-HIT with a 30% sequence identity threshold to eliminate redundancy and potential bias, ensuring a diverse representation of ATP-binding proteins for model development and evaluation as listed in Table 1.

**Table 1:** Statistics of the Survey Dataset

	data set	protein sequences	similarity <30%	ATP binding residues	non-binding residues
training	ATP-388	388	322	4706	117,599
	ABC Transporter	178	173	1794	132,962
	total	566	500	6484	265,771
testing	CFTR	115	29	298	24,878

In addition to data set statistics, we examined the sequence length distributions of the ATP-388 and ABC transporter data sets. The analysis confirmed that proteins in the ABC transporter data set generally have longer sequences than those in ATP-388. This reflects the greater structural and functional complexity of ABC transporters, which often include extended transmembrane and nucleotide-binding domains. Such length differences further justify the inclusion of ABC transporter proteins in the transfer learning stage, as they provide CFTR_TL with richer sequence contexts that are more representative of CFTR.

### Feature Extraction

To predict ATP-binding sites in CFTR, we utilized protein language models (PLMs) from the ProtTrans^7^ series. These models, adapted from natural language processing, are designed for protein sequence analysis through unsupervised pretraining on large protein data sets. During this training, the models capture both structural and functional information, allowing them to generate high-dimensional embeddings that reflect subtle sequence variations and contextual relationships critical to protein function.

ProtTrans, built on the Transformer architecture, was pretrained on the UniRef50 protein sequence collection, enabling it to identify common patterns across diverse protein families. In this study, we specifically used the ProtTrans-T5-XL-U50 variant, which generates embeddings of up to 1024 dimensions. These high-dimensional representations go beyond basic amino acid sequences by incorporating detailed structural and functional insights. This comprehensive understanding allows ProtTrans-T5-XL-U50 to outperform traditional methods that rely on manually selected features, especially for complex tasks such as ATP-binding site prediction. By recognizing patterns within the sequence context, this model effectively captures functional domains and interaction sites, leading to more accurate predictions of ATP-binding sites in CFTR.

### Multiple Window Scanning Deep Learning Networks Architecture

We employed a multiple window scanning technique to capture sequence patterns at different scales. Protein sequences are inherently linear, and their biochemical information lies in the sequential order of amino acids rather than in a two-dimensional spatial arrangement. A conventional 2D-CNN, which is optimized for image-like data, would introduce artificial spatial assumptions and redundant parameters that are unnecessary for one-dimensional sequences. Likewise, a traditional single-window 1D-CNN can capture only a fixed local context and often fails to integrate signals spanning different sequence ranges.

Our multiwindow scanning design overcomes these limitations by applying several convolutional filters of different window sizes in parallel to the high-dimensional sequence embeddings. This allows the network to detect short local motifs as well as broader contextual dependencies within the same framework. For each window, the convolutional outputs are computed over overlapping regions, followed by max-pooling to retain the most informative features. The pooled vectors from all windows are concatenated and passed to fully connected layers for final prediction. Window lengths from 2 to 12 were systematically tested, and the combination of 6, 8, 10, and 12 residues achieved the best balance between local detail and global context, leading to the highest predictive accuracy while maintaining computational efficiency.

### Performance Evaluation

The predictive model was trained using a carefully prepared training data set, with hyperparameter tuning performed to optimize performance on the validation set. The model’s effectiveness was assessed using standard evaluation metrics such as sensitivity, specificity, accuracy, Matthews correlation coefficient (MCC), and the area under the receiver operating characteristic curve (AUC). Sensitivity and specificity were particularly important in evaluating the model’s ability to accurately distinguish ATP-binding and nonbinding residues in CFTR.

1 $$\mathrm{Sensitivity}=\left(\frac{\mathrm{TP}}{\mathrm{TP}+\mathrm{FN}}\right)$$

2 $$\mathrm{Specificity}=\left(\frac{\mathrm{TN}}{\mathrm{TN}+\mathrm{FP}}\right)$$

3 $$\mathrm{Accuracy}=\left(\frac{\mathrm{TP}+\mathrm{TN}}{\mathrm{TP}+\mathrm{TN}+\mathrm{FP}+\mathrm{FN}}\right)$$

4 $$\mathrm{MCC}=\left(\frac{\left(\mathrm{TP}\times \mathrm{TN}\right)-\left(\mathrm{FP}\times \mathrm{FN}\right)}{\sqrt{\left(\mathrm{TP}+\mathrm{FP}\right)\left(\mathrm{TP}+\mathrm{FN}\right)\left(\mathrm{TN}+\mathrm{FP}\right)\left(\mathrm{TN}+\mathrm{FN}\right)}}\right)$$

These metrics are based on the classification of predictions into four categories: True Positives (TP), True Negatives (TN), False Positives (FP), and False Negatives (FN). Sensitivity, or recall, measures the proportion of actual ATP-binding residues correctly identified by the model, while specificity evaluates how well the model classifies nonbinding residues. Accuracy represents the overall proportion of correct classifications, regardless of class. MCC provides a balanced evaluation of the model’s performance by considering all four categories, with values ranging from −1 (complete disagreement between predictions and true values) to +1 (perfect prediction), where 0 indicates performance no better than random. AUC, the area under the ROC curve, reflects the model’s ability to distinguish between binding and nonbinding residues across different classification thresholds. An AUC of 1 indicates perfect discrimination, while a value of 0.5 represents random classification. Our model’s ability to predict ATP-binding sites in CFTR was evaluated using these metrics, with AUC being the primary criterion due to its ability to summarize performance across varying thresholds.

The overall computational pipeline of CFTR_TL is summarized in the following schematic to illustrate the major steps from sequence input to ATP-binding site prediction (Figure 3).

**Figure 3:**
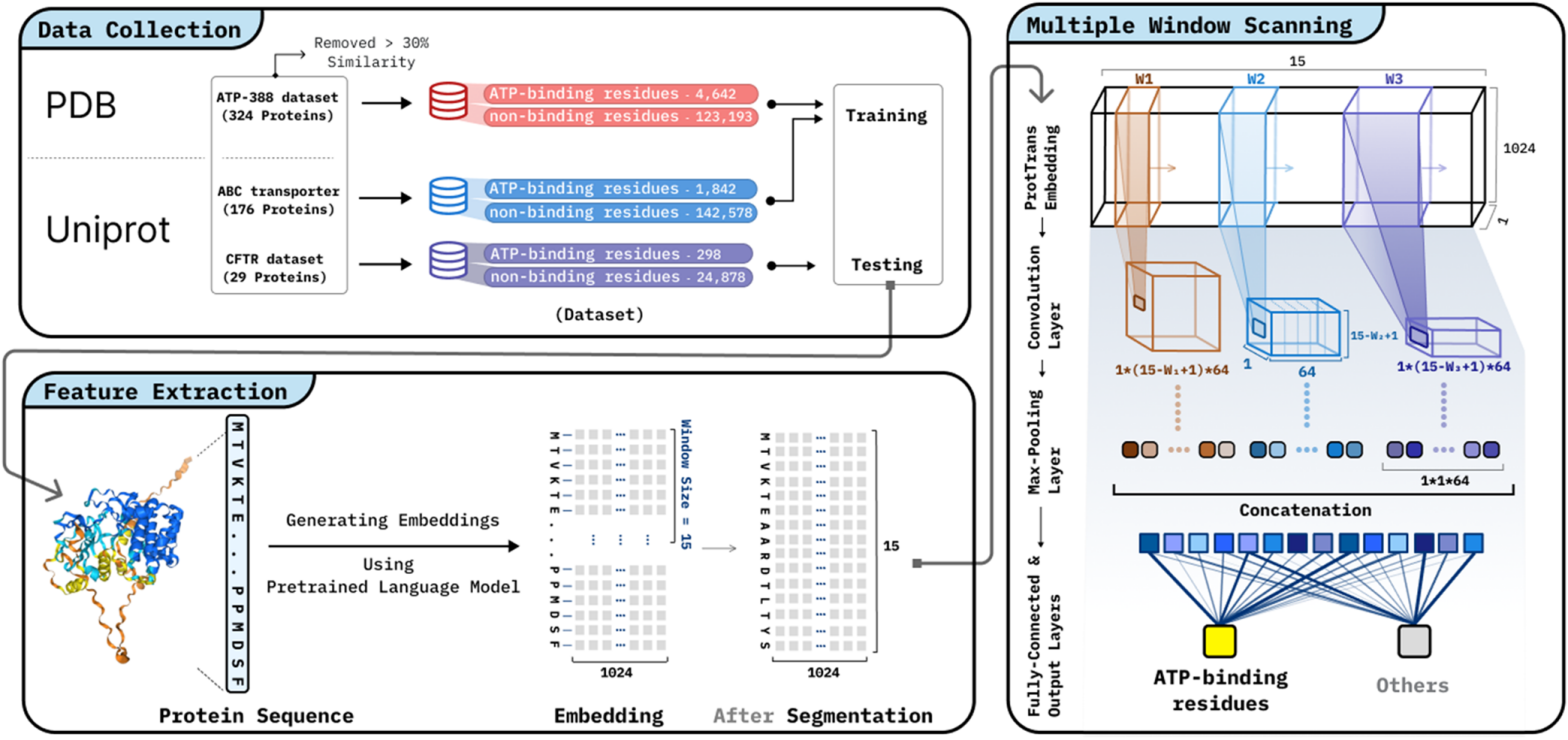
Workflow for ATP binding site in CFTR Protein prediction model.

## Results

### Comparison of Performance with Different Sizes of Single Windows

We began by examining the effect of various single window sizes on the model’s performance. As detailed in Table 2, we noted a steady increase in AUC values as the window size expanded, with the highest AUC reaching 0.9860 at a window size of 10. This pattern indicates that larger windows are more effective at capturing sequence features related to ATP-binding sites, as they provide a wider context, offering a more complete representation of the sequence information surrounding the binding sites. However, beyond a certain window size, the performance improvements level off, suggesting that excessively large windows may introduce irrelevant sequence data, which can negatively impact the accuracy of predictions.

**Table 2:** Comparison of Performance with Different Sizes of Single Windows

single window sizes	sensitivity	specificity	accuracy	MCC	AUC
2	0.9458	0.9238	0.9243	0.4570	0.9807
4	0.9422	0.9299	0.9302	0.4719	0.9821
6	0.9432	0.9378	0.9380	0.4955	0.9840
8	0.9450	0.9414	0.9415	0.5082	0.9850
10	0.9458	0.9413	0.9414	0.5092	0.9852
12	0.9381	0.9382	0.9382	0.4947	0.9839

Thus, it becomes crucial to balance window size to ensure that the model captures essential information without being overwhelmed by extraneous data. Optimizing the window size is a key factor in enhancing model performance, as it ensures the model extracts relevant sequence features while minimizing noise, leading to more accurate predictions.

### Comparison of Performance with Different Combinations of Windows

Next, we evaluated the model’s performance using various combinations of window sizes. The selection of windows was guided by the AUC rankings from Table 2. As shown in Table 3, the combination of window sizes 6, 8, 10, and 12 yielded the highest AUC value of 0.9860. This highlights the effectiveness of a multiscale approach, where different window sizes capture complementary sequence features, leading to more accurate and well-rounded predictions.

**Table 3:** Comparison of Performance with Different Combinations of Windows

window combinations	sensitivity	specificity	accuracy	MCC	AUC
[4, 6 ]	0.9468	0.9341	0.9344	0.4854	0.9841
[4, 6, 8 ]	0.9505	0.9362	0.9365	0.4941	0.9855
**[4, 6, 8, 10 ]**	**0.9493**	**0.9418**	**0.9420**	**0.5118**	**0.9862**
[4, 6, 8, 10, 12 ]	0.9437	0.9406	0.9407	0.5063	0.9858
[2, 4, 6, 8, 10, 12 ]	0.9460	0.9425	0.9426	0.5121	0.9857
[6, 8, 10, 12 ]	0.9443	0.9438	0.9438	0.5158	0.9860
[8, 10, 12 ]	0.9429	0.9398	0.9398	0.5014	0.9854
[10, 12 ]	0.9519	0.9331	0.9336	0.4855	0.9857

The success of these multiwindow combinations emphasizes the complexity of ATP-binding sites, which likely involve interactions across multiple sequence scales. While a single window size can capture information at one specific scale, combining multiple windows allows the model to integrate both local and broader sequence features. This enables the identification of specific motifs while also recognizing larger patterns, significantly improving the model’s ability to predict ATP-binding sites.

### Comparison of Performance with Different Filter Numbers

To determine the optimal number of filters in the convolutional layers, we tested model performance using different filter sizes, as shown in Table 4. The model achieved its best performance with 64 filters, reaching an AUC of 0.9863. Increasing the number of filters beyond this point did not yield further improvements and, in some instances, caused a slight performance decline, likely due to overfitting.

**Table 4:** Comparison of Performance with Different Filter Numbers

filters sizes	sensitivity	specificity	accuracy	MCC	AUC
128	0.9558	0.9340	0.9346	0.4918	0.9860
256	0.9484	0.9427	0.9428	0.5151	0.9862
**512**	**0.9547**	**0.9371**	**0.9375**	**0.4987**	**0.9863**
1024	0.9499	0.9411	0.9413	0.5093	0.9860

This result highlights a fundamental principle in deep learning model design: balancing model complexity with generalization ability. While increasing the number of filters can allow the model to extract more detailed features from the input data, which can enhance training performance, excessive complexity can cause the model to overfit the training data, leading to diminished accuracy on unseen data. Therefore, selecting an optimal number of filters is key to ensuring the model captures meaningful patterns without sacrificing its ability to generalize to new data.

### Comparison of Performance with Different Imbalance Handling

To assess the influence of class imbalance handling on predictive performance, we compared CFTR_TL trained without resampling and with three oversampling strategies: Synthetic Minority Oversampling Technique (SMOTE), Adaptive Synthetic Sampling (ADASYN), and random oversampling. As shown in Table 5, oversampling produced only minor gains in sensitivity and Matthews correlation coefficient (MCC), while the area under the ROC curve (AUC) remained essentially unchanged (all >0.985). Among the tested methods, ADASYN achieved the highest sensitivity and MCC, but the overall improvements were marginal, indicating that CFTR_TL maintains strong discriminative power and is intrinsically robust to class imbalance even without explicit resampling.

**Table 5:** Comparison of Performance with Different Imbalance Handling

	sensitivity	specificity	accuracy	MCC	AUC
None	0.9452	0.9525	0.9524	0.4284	0.9864
SMOTE	0.9623	0.9376	0.9410	0.4536	0.9866
ADASYN	0.9652	0.9335	0.9390	0.4623	0.9865
RANDOM	0.9581	0.9289	0.9370	0.4521	0.9850

### Comparison of Performance with Different Training Data

The Table 6 shows that combining the ATP-388 data set (general ATP-binding proteins) and the ABC transporter data set (functionally related to CFTR) yields a significantly better model for predicting ATP binding sites in CFTR than using either data set alone. The combined data set (ATP-388 + ABC) improves both sensitivity (0.9521) and accuracy (0.9389) substantially compared to either the ATP-388 data set (0.9281 sensitivity and 0.9237 accuracy) or the ABC transporter data set (0.8288 sensitivity and 0.9069 accuracy). This indicates that the combined data set provides a more comprehensive and nuanced understanding of the sequence features associated with ATP binding sites, and particularly those features specific to the ABC transporter family. The improvement in the combined model suggests that the unique characteristics of CFTR’s ATP-binding site are better captured and generalized by learning from both general ATP-binding proteins and those structurally and functionally related to it within the ABC family.

**Table 6:** Comparison of Performance with Different Training Data

	sensitivity	specificity	accuracy	MCC	AUC
ATP-388	0.9281	0.9236	0.9237	0.3393	0.9703
ABC Transporter	0.8288	0.9080	0.9069	0.2744	0.9344
**ATP-388 + ABC**	**0.9521**	**0.9387**	**0.9389**	**0.4858**	**0.9804**

### Comparison of Performance with Different Pretrained Language Model

To evaluate the adaptability of CFTR_TL to different protein language model embeddings, we further compared its performance using features derived from ProtTrans, TAPE, ESM-2, and ProtBERT (Table 7). Among these models, **ProtTrans achieved the best overall performance** with the highest AUC (0.9863) and MCC (0.4987). The newer **ESM-2 model exhibited very competitive results** (AUC = 0.9852, MCC = 0.4602), outperforming both TAPE (AUC = 0.9650, MCC = 0.4209) and ProtBERT (AUC = 0.9821, MCC = 0.4207). Although ProtTrans provided a slight advantage in most metrics, the strong performance of ESM-2 underscores the potential of next-generation protein language models to further enhance sequence-based ATP-binding site prediction as these models continue to evolve.

**Table 7:** Comparison of Performance with Different Pre-Trained Language Model

model	sensitivity	specificity	accuracy	MCC	AUC
ProtTrans	0.9547	0.9371	0.9375	0.4987	0.9863
**TAPE**	0.8812	0.9415	0.9325	0.4209	0.9650
**ESM-2**	0.9350	0.9553	0.9426	0.4602	0.9852
**ProtBERT**	0.9482	0.9324	0.9350	0.4207	0.9821

### Comparison of Performance with Different Classifiers

The results show that our mCNN model outperforms traditional methods like SVM, KNN, and RF in ATP-binding site prediction. mCNN achieves the highest sensitivity (0.9547) and a strong AUC (0.9863), making it more reliable at identifying true ATP-binding residues. While KNN and RF have high specificity, their sensitivity is significantly lower, making them less effective overall as listed in Table 8 and illustrated in Figure 4. mCNN provides the best balance across all metrics, ensuring accurate and comprehensive predictions.

**Figure 4:**
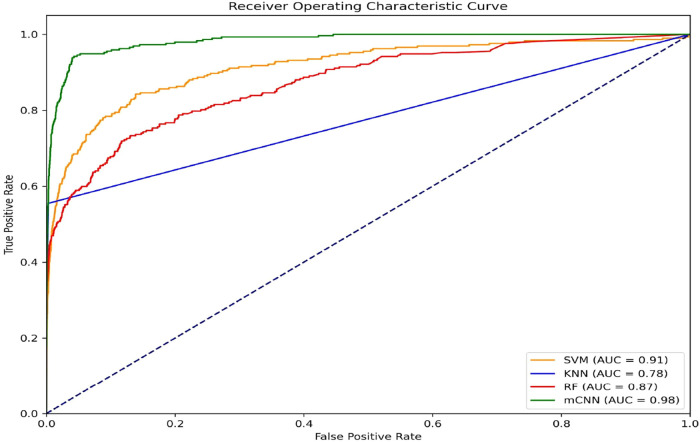
ROC Curve for the performance of predicting ATP Binding Site in CFTR with different Classifiers.

**Table 8:** Comparison of Performance with Different Classifiers

	sensitivity	specificity	accuracy	MCC	AUC
SVM	0.8836	0.8702	0.9348	0.2447	0.9433
KNN	0.5240	0.9972	0.9911	0.6035	0.9057
RF	0.0000	1.0000	0.9872	0.0000	0.9251
**mCNN**	**0.9547**	**0.9371**	**0.9375**	**0.4987**	**0.9863**

### Comparison of Performance with Previous Works

Comparing the performance of CFTR_TL with previous methods reveals significant improvements in predicting ATP-binding sites in CFTR. Existing methods like NsitePred, TargetATPsite, and TargetNUCs show relatively low sensitivity (ranging from 0.27 to 0.8), indicating a substantial limitation in correctly identifying true ATP-binding residues. While these methods often exhibit high specificity (typically above 0.97), the trade-off leads to lower overall accuracy and MCC. Even the more sophisticated ProtT5_CNN model, while achieving decent accuracy, falls short of CFTR_TL ’s superior sensitivity of 0.9452 and an impressive AUC of 0.9863 as listed in Table 9 and illustrated in Figure 5. This demonstrates that CFTR_TL effectively balances high sensitivity with acceptable specificity, resulting in a markedly improved overall prediction performance for CFTR compared to prior approaches.^16–18^

**Figure 5:**
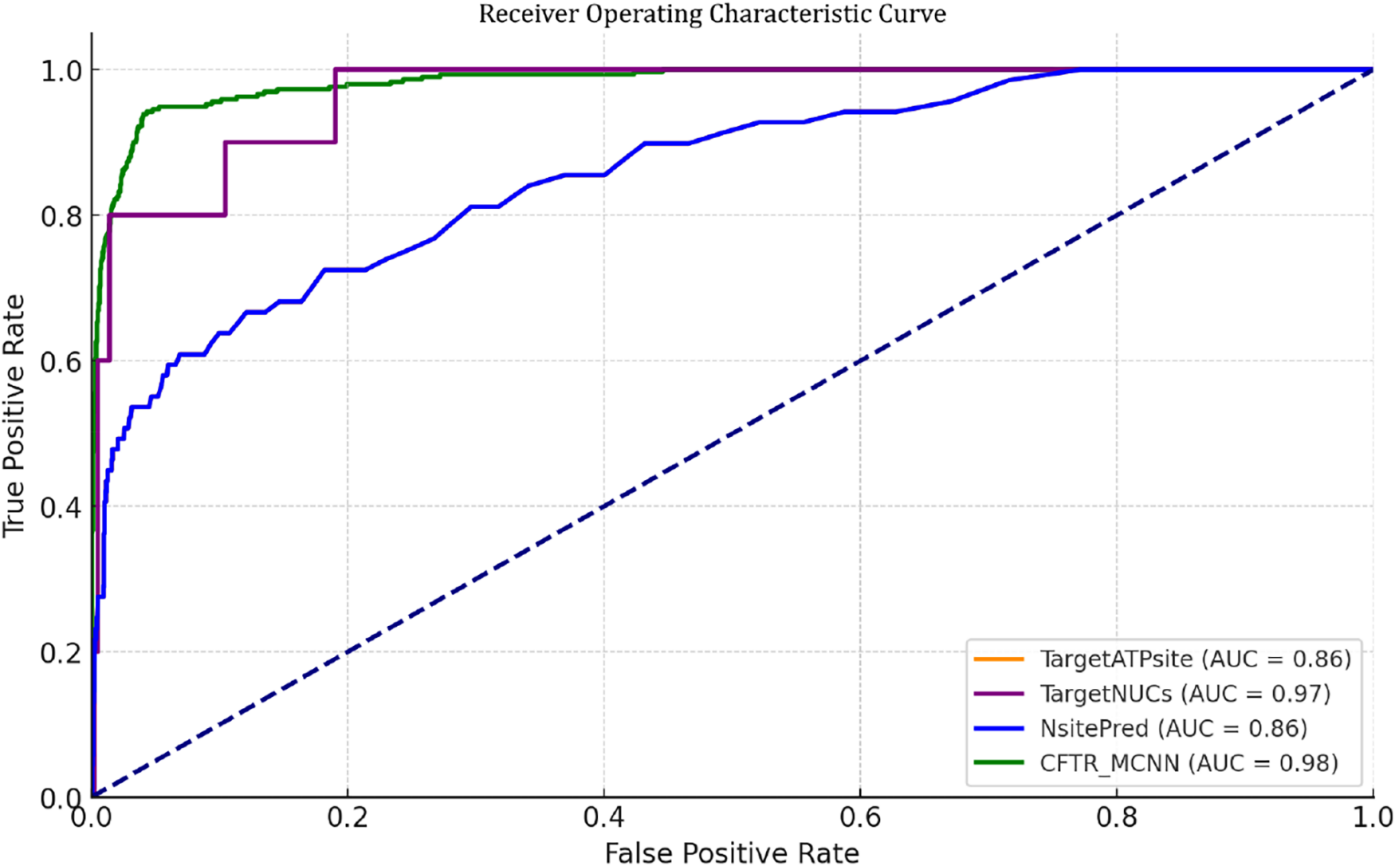
ROC Curve for the performance of predicting ATP binding site in CFTR with different ATP-binding site models.

**Table 9:** Comparison of Performance with Previous Works

methods	sensitivity	specificity	accuracy	MCC	AUC
NsitePred	0.2754	0.9907	0.9751	0.3179	0.8576
targetATPsite	0.2738	0.9749	0.9539	0.2393	0.8073
targetNUCs	0.8000	0.9786	0.9745	0.6022	0.9653
**our method**	**0.9452**	**0.9525**	**0.9524**	**0.4284**	**0.9863**

## Conclusions

This study presents CFTR_TL, a novel deep learning model designed for accurate prediction of ATP binding sites within the CFTR protein. The model leverages a multiwindow CNN architecture, enhanced by pretrained protein language models (ProtTrans embeddings), and a transfer learning strategy. Traditional ATP binding site prediction methods often rely on generic sequence motifs or manually crafted features, which may fail to fully capture the intricate functional dependencies of CFTR. In contrast, CFTR_TL effectively combines deep hierarchical feature representations, leveraging the power of ProtTrans embeddings to incorporate evolutionary and biochemical information from vast protein databases. The transfer learning approach allows the model to fine-tune its predictions based on CFTR-specific data, improving its adaptability and precision. This approach integrates features from diverse scales, capturing both local and global sequence contexts associated with ATP binding.

Evaluation results demonstrate that CFTR_TL achieves significantly higher performance compared to existing prediction methods. Critically, the model exhibits high accuracy, sensitivity, and area under the ROC curve (AUC), surpassing previous models, especially in correctly identifying true ATP-binding residues. As illustrated in Figure 6 These results suggest that CFTR_TL can seamlessly integrate emerging protein language models, providing a promising avenue for future performance improvements. By addressing the unique structural and functional complexities of CFTR, CFTR_TL sets a new benchmark in ATP binding site prediction, offering an invaluable tool for researchers exploring CFTR dysfunction in cystic fibrosis. The enhanced accuracy and reliability of CFTR_TL not only contribute to a deeper molecular understanding of CFTR-related pathologies but also hold promising implications for the development of targeted therapeutics aimed at restoring CFTR function in cystic fibrosis patients.

**Figure 6:**
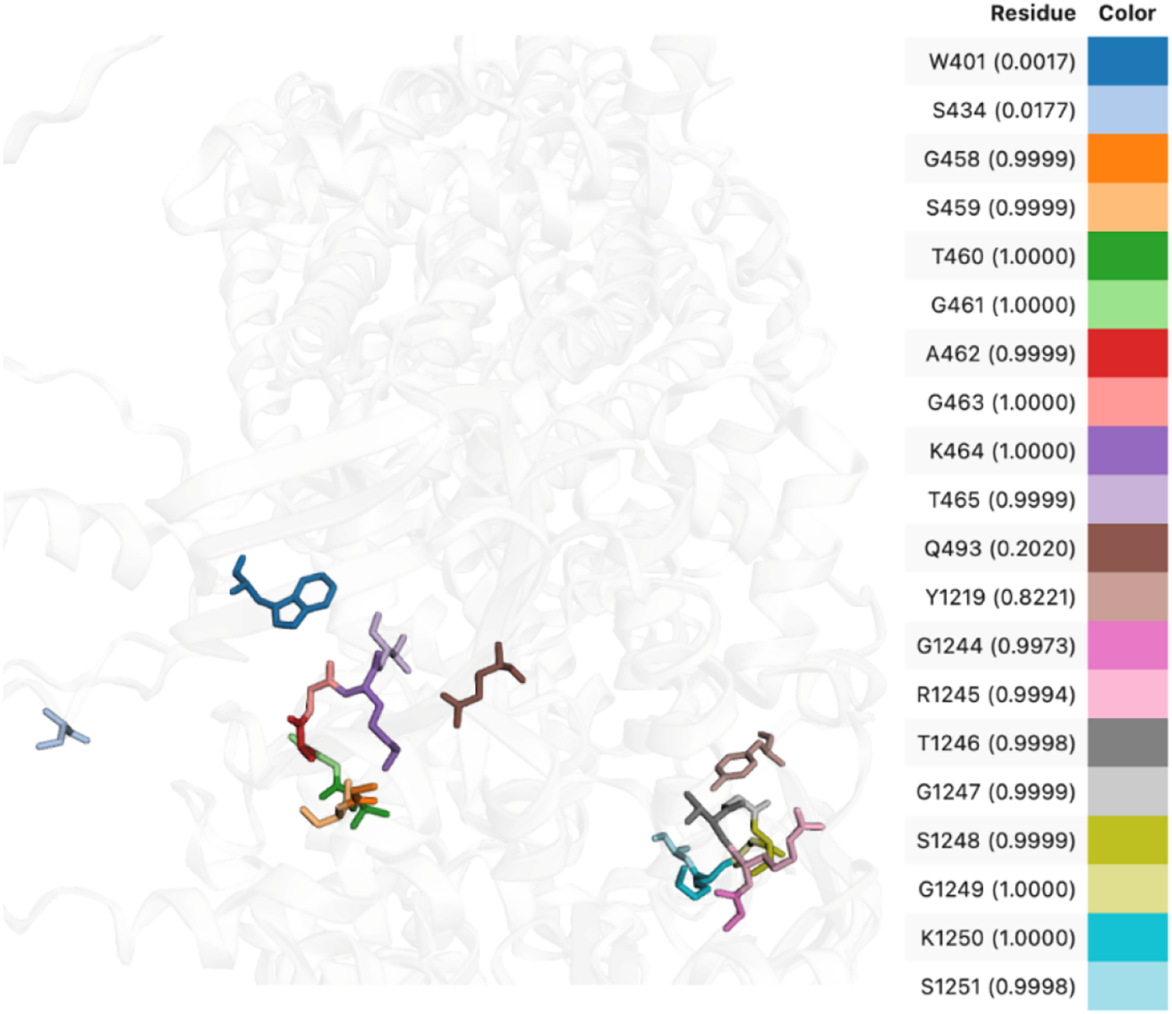
Predicted ATP-binding residues in CFTR mapped onto the 3D structure. Each colored stick represents a residue predicted by CFTR_TL, with the color corresponding to the residue identity and the accompanying value indicating the predicted binding probability.

A crucial aspect of CFTR_TL ’s success stems from its training on a combined data set comprising general ATP-binding proteins (ATP-388) and ABC transporter proteins (which are structurally and functionally related to CFTR). This combination is essential for capturing both general and specific characteristics of ATP-binding sites. As illustrated in Table 5, the performance improvement in using the combined data set (0.9804 AUC) over using either data set individually (0.9703 and 0.9344 AUC respectively) showcases the value of incorporating both general and related protein sequence information. This strategy allows CFTR_TL to learn more comprehensive and contextually appropriate features, ultimately leading to more accurate CFTR-specific predictions.

The framework introduced in CFTR_TL not only advances ATP binding site prediction within the CFTR protein but also provides a generalizable approach that can be adapted to other protein families with distinct structural and functional characteristics. By leveraging a combination of multiwindow CNN architectures, deep contextual embeddings from PLMs, and transfer learning, this methodology can be extended to predict functional sites in other complex proteins, such as ion channels, transporters, and enzymatic regulators. Many proteins exhibit unique conformational dynamics, binding affinities, and sequence variability, making general ATP binding site prediction challenging. However, CFTR_TL demonstrates that by customizing deep learning architectures to a protein’s specific biochemical properties, substantial improvements in predictive accuracy and reliability can be achieved. Future research could focus on investigating the effect of different window combination strategies or refining the transfer learning approach to further optimize performance. The code and data will aid researchers in replicating and expanding this model for other protein prediction tasks.

In conclusion, CFTR_TL represents a significant advancement in predicting ATP binding sites in CFTR, showcasing the substantial benefits of combining data sets and leveraging a multiscale, context-aware approach. This model’s performance improvement underscores its practical utility for understanding CFTR function, advancing cystic fibrosis research, and potentially driving therapeutic advancements. The findings highlight the potential of combining protein sequence data with transfer learning and specialized architectures for enhanced protein prediction, a critical area for advancing our understanding of biological systems.

## Key Points

1. CFTR-Specific ATP Binding Site Prediction: The study addresses the critical need for accurate prediction of ATP binding sites within the CFTR protein, which is essential for understanding cystic fibrosis and developing targeted therapies.
2. Transfer Learning: CFTR_TL leverages transfer learning by first training a base model on a general ATP-binding data set and then transfer learning it on an ABC transporter-enriched data set, exploiting their functional similarity to CFTR. This approach allows the model to specialize its predictive capabilities for this unique protein family.
3. ProtTrans Embeddings Enhance Performance: The utilization of ProtTrans embeddings, derived from pretrained protein language models, significantly improves the model’s ability to capture rich sequence information and contextual patterns, leading to superior performance compared to traditional methods.
4. Multi-Window CNN Captures Multi-Scale Features: Employing a multiwindow CNN architecture enables the model to integrate features across multiple scales, capturing both local motifs and broader sequence contexts that contribute to ATP binding specificity. This multiscale analysis further enhances the accuracy and reliability of predictions.
5. Superior Performance and Generalizability: CFTR_TL demonstrates significantly higher accuracy (AUC of 0.9863) compared to existing ATP binding site prediction methods, particularly for CFTR. Furthermore, the framework presented offers a generalizable approach for tailoring prediction models to other specific protein families, potentially improving predictions in various biological contexts.

## Data Availability

In addition, the code and data for this work are available at: https://github.com/B1607/CFTR_TL.
